# Numerical study of copper antimony sulphide (CuSbS_2_) solar cell by SCAPS-1D

**DOI:** 10.1016/j.heliyon.2024.e26896

**Published:** 2024-02-22

**Authors:** Nancy Obare, Wycliffe Isoe, Amos Nalianya, Maxwell Mageto, Victor Odari

**Affiliations:** a Department of Physics, Masinde Muliro University of Science and Technology, P.O Box 190-50100, Kakamega, Kenya; b Materials Research Society of Kenya, P.O. Box 15653-00503, Nairobi, Kenya

**Keywords:** CuSbS_2_, SCAPS-1D, Thin film, Simulation

## Abstract

Copper antimony sulphide thin films are promising, less toxic, and more absorbent material in the world, and they would be good to be applied in photovoltaic energy production. To better operations of copper antimony sulphide (CuSbS_2_) photovoltaic cells, this paper uses a solar cell capacitance simulator (SCAPS-1D) to simulate and analyze photovoltaic properties. This article examines different thicknesses of fluorine-doped tin oxide (FTO), cadmium sulphide (CdS), carbon (C), and CuSbS_2_, as well as the defect and dopant concentration in the CuSbS_2_ photoactive layer of the photovoltaic cell structure glass/FTO/n-CdS/p-CuSbS_2_/C/Au. Optimum thicknesses of CuSbS_2_ is 300 nm, carbon hole transport layer (HTL) is 50 nm, and for n-CdS electron transport layer (ETL) is 100 nm, giving open circuit Voltage (Voc) of 0.9389 V, short circuit current density (Jsc) of 28.32 mA/cm^2^, fill factor (FF) of 60.8% and solar cell efficiency of 16.17%. The increase in defects causes a decrease of carrier lifetime resulting in to decrease in diffusion length and the optimum absorber layer doping concentration was found to be 10^18^ cm^−3^.

## 1 Introduction

The thin films based on chalcogenide have gained much interest from researchers as a promising material for application in photovoltaics. The highest efficiencies of thin films photovoltaic cells under the chalcogenide photoactive material like cadmium telluride (CdTe) and copper indium gallium selenide (CIGS) are 22.9% and 23.1% respectively [1]. Perhaps, the toxic nature of cadmium and the high cost of indium, telluride, and gallium have been major limiting factors for the production of these solar cells [2]. Copper antimony sulphide is a ternary layer semiconductors with low toxicity, an optical bandgap of about 1.5 eV, and an absorption coefficient of above 10^5^ cm^−1^ making it stable and suitable for large-scale photovoltaic power [3]. Several experimental investigations have been done on CuSbS_2_ photovoltaic cells. The efficiency of 3.13% was attained by the fabrication of copper antimony sulphide-based photovoltaic cells from electrodeposited copper/antimony stacked layers followed by sulphurization at 450 °C in hydrogen sulphide gas for 30 min [4]. Spin coated thin films of CuSbS_2_ thin films by Cu–Sb–S stock solution on fluorine-doped tin oxide substrates were reported by Yang et al., followed by multiple steps of annealing, and obtained an efficiency of 0.5% by simulation of the material using photovoltaic cell design FTO/CuSbS_2_/CdS/ZnO/ZnO:Al/Au. The obtained low power conversion efficiency was a result of low open-circuit voltage at the interface resulting from severe recombination loss between copper antimony sulphide and Cadmium sulphide layers [5]. Macias et al. studied the solar cell architecture SnO_2_:F/CdS/Sb_2_S_3_/CuSbS_2_/C:Ag and found an efficiency of 0.66% because of low open-circuit voltages [6]. Olopade investigated the effect of CdS, InS, ZnSe, and ZnS thin films material as buffer layers in copper antimony sulphide photovoltaic cells through computational using SCAPS-1D and reported an efficiency of 3.78% of the photovoltaic cell structure ZnO:Al/CdS/CuSbS_2_/Mo. By optimizing the indium sulphide buffer layer, the efficiency improved to 3.88% indicating that, it was an appropriate buffer layer although the cost of indium which is relatively expensive limits its use [7]. The effect of low open circuit voltage has a major challenge hindering the power conversion of this photovoltaic cells [8]. Also, by considering the solar cell structure In_2_S_3_/CdS/CuSbS_2_/Mo, an efficiency of 2.99% was attained which is due to a cliff-like conduction band [1]. Sadanand et al. did a comparison study between CZTS, CuSbS_2,_ and CuSbSe_2_ using solar cell architecture ITO/ZnS/CuSbS_2_/Mo and obtained an efficiency of 17.3% while using CuSbS_2_ as absorber layer [9], indicating a promising result for an alternative material which is relatively cheap, earth-abundant and less toxic [10].

In device fabrication of solar cells, the choice of material for each layer has an impact on the workings of the cell. This research paper uses SCAPS-1D software developed by the University of Gent to perform numerical simulation of CuSbS_2_ photovoltaic cells with the architecture glass/FTO/n-CdS/p-CuSbS_2_/C/Au [11,12]. This project aims further improve its photovoltaic performance. This is done by determining optimal thickness of the hole transport layer (HTL), electron transport layer (ETL), and absorber layers and examining the effects of dopant concentration and defects on the photoactive layer.

## 2 Materials and methods

SCAPS-1D software developed by the University of Gent uses this program to solve semiconductor equations numerically under a steady state in one dimensional using Poisson's and continuity equations. The charges density space (ρ) and electric field (E) of a heterojunction given by the Poisson equation is given by the following relationship in equation (1) [13].

[1] $$\frac{d^{2}\varphi }{dx^{2}}=\frac{q}{\varepsilon_{o}\varepsilon_{r}}\left[N_{D}-N_{A}\right]+\left(p_{x}-n_{x}\right)+\left(P_{p}-p_{n}\right)$$

where ψ is electrostatic potential, q is charge, N_D_ (N_A_) is donor or acceptor ionized density, n(p) is densities of electron or hole, $\varepsilon_{r}$ is the medium's relative permittivity, and P_p_/P_n_ is possible defects density of acceptors or donors. The continuity equation for a steady state of electron and hole is given in equations [2], [3]):

[2] $$\frac{\partial j_{n}}{\partial x}+G-U_{n}\left(n,p\right)=0$$

[3] $$-\frac{\partial j_{p}}{\partial x}+G-U_{p}\left(n,p\right)=0$$

J_n_, and j_p_ are the current densities of electron and hole, G is the generation rates of electrons-holes, and U (n,p) is net recombination rates. Equations [4], [5]) gives electrons and holes densities as:

[4] $$j_{n}=qn\mu_{n}E+qD_{n}\frac{\partial p}{\partial x}$$

[5] $$j_{p}=qp\mu_{p}E-qD_{p}\frac{\partial p}{\partial x}$$

where q is the charge $\mu_{n,\left(p\right)}$, is the mobility of electrons or holes and D_n,(p)_ is the diffusion coefficient of an electron or a hole.

In this study, CuSbS_2_ was used as a photoactive material in the cell structure glass/FTO/n-CdS/p-CuSb_2_/C/Au to elaborate its optoelectronic properties and application in photovoltaics. CdS and C have been used as HTL and ETL, respectively. The back contact of gold with a work function 5.1 eV was used as a cathode. All simulations were done at a constant temperature of 300K, and incident power of 1000 W/m^2^ of the spectrum of AM 1.5G. we introduced one defect mechanism of single level (neutral) [13], with the single energetic distribution of 0.6 eV above the highest E_V,_ for close examination of recombination and their impact on photovoltaic cell workings [14].

### 2.1 Material Parameters and numerical Model for the copper antimony sulphide solar cell

**Fig. 1:**
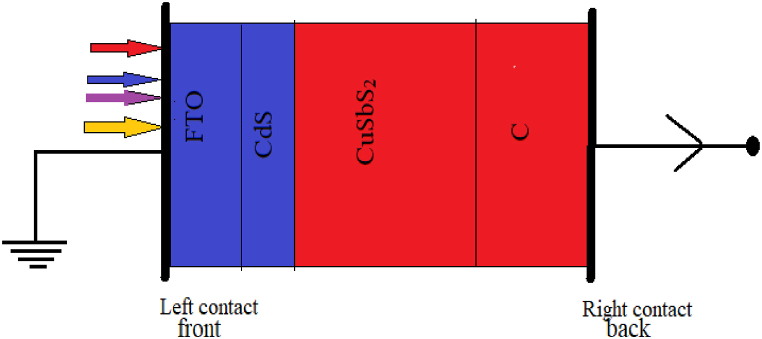
CuSbS_2_ solar cell architecture.

The structure and characterizing of the CuSbS_2_ model were simulated by SCAPS-1D version 3.3.07 [11]. The design of the photovoltaic cell was FTO/CdS (ETL)/CuSbS_2_(Absorber)/C(HTL)/Au (back contact) with CdS/CuSbS_2_ and CuSbS_2_/C interfaces having neutral defect layers as described in Fig. 1. Optimization of the solar cell structure of each layer is important for the performance of the photovoltaic cell, Fluorine-doped tin oxide is an n-type material with a wide bandgap of 3.5 eV and a work function of 4.4 eV was preferred as an anode (front contact) similar procedure to the work reported by Hossain and his coworkers [15,]. Cadmium sulphide was used as an electron transport layer hence preventing holes from reaching the front contact. FTO was preferred to ITO because it has special characteristics in chemical inertness, upon heating it is stable, hard mechanically, a good conductor, relatively cheap and its sheet resistance remains constant during sintering [16]. In addition, tin oxide films in the visible range are highly transparent. Table 1 shows layer values used in the study which were obtained from other reported works, [7,15].

**Table 1:** Simulation parameters for CuSbS_2_ photovoltaic cell.

Photovoltaic cell parameter	FTO	CuSbS_2_	CdS	C
Bandgap, $E_{g}$ (eV)	3.5	1.5	2.42	3
Thickness, d (μm)	0.1 (varied)	0.3 (varied)	0.01	0.35
Electron affinity, χe (eV)	4	3.85	4.5	2.45
Relative dielectric permittivity,	9	14.6	10	3
NC effective density (${\text{cm}}^{-3}$)	2.2 $\times 10^{18}$	2.2 $\times 10^{18}$	2.2 $\times 10^{19}$	2.2 $\times 10^{18}$
NC effective density (${\text{cm}}^{-3}$)	1.8 $\times 10^{19}$	1.8 $\times 10^{19}$	1.8 ×$10^{19}$	1.8 $\times 10^{19}$
Thermal velocity of electron (cm/s)	1.8 $\times 10^{7}$	1.8 $\times 10^{7}$	1.0 ×$10^{7}$	1.0 $\times 10^{7}$
Thermal velocity of a hole (cm/s)	1.0 $\times 10^{7}$	1.0 $\times 10^{7}$	1.0 $\times 10^{7}$	1.0 $\times 10^{7}$
Mobility of electron, μ_n_ (${\text{cm}}^{2}/\text{Vs}$)	20	60	100	2× $10^{-4}$
Hole mobility, μ_p_ ($cm^{2}/Vs$)	10	20	25	2× $10^{-4}$
Donor density o, N_D_ (${\text{cm}}^{-3}$)	1 $\times 10^{18}$	–	1 $\times 10^{17}$	–
N_A_ Acceptor density	–	1 × 10^18^	–	2 × 10^18^

### 2.2 Alignment of band structure

**Fig. 2:**
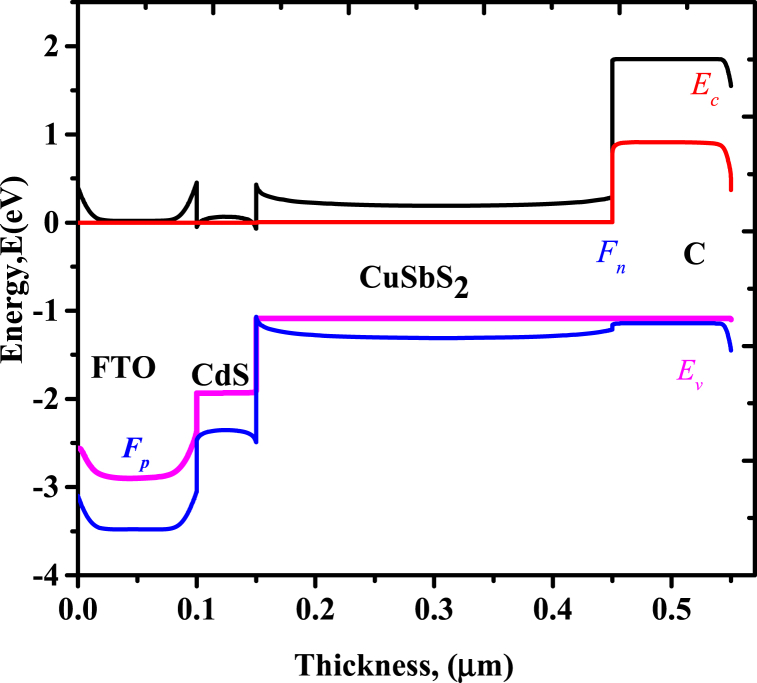
Energy band diagram.

The operation of the photovoltaic cell devices depends on the alignment of band structure for the transportation of photogenerated carriers and it is also affected by the different electron affinity between buffer material and absorber material [17,18]. The optimized band diagram is shown in Fig. 2.

Where E_c,_ E_v,_ F_p,_ and F_n_ are conduction energy, valence energy, and Quasi-Femi energy for electron and hole respectively. The electron affinity and photoactive layer give the nature of heterointerface band, [19]. From the alignment, there is a spike-like conduction bands offset at CdS implying an inbuilt potential barrier to photogenerated electrons [20]. This is the limiting factor because, a cliff-like conductions bands offsets rejects electron coming from a forward biasing, faces hindrance at CdS/CuSbS_2_ interface and recombination between majority charge carriers rises through defects available at the interface leading to a decrease of open circuit voltage.

## 3 Result and discussion

### 3.1 Impact of fluorine-doped tin oxide thickness

**Fig. 3:**
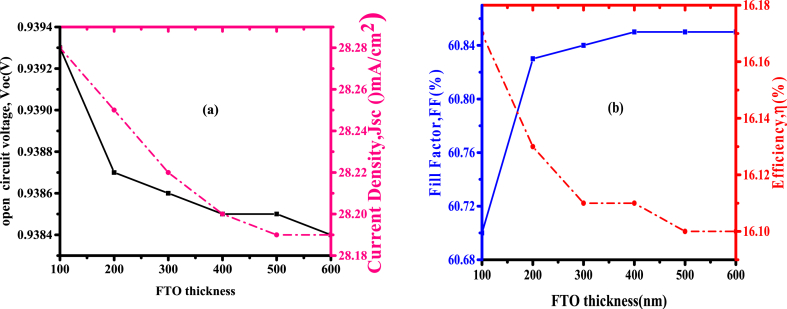
Variation of (a) *Voc* and *Jsc* (b) *FF* and efficiency with FTO thickness.

Transparent conductive oxide (TCOs) are used as a front electrode and as a backside reflector [21]. The working of the copper antimony sulphide cell was studied from the variation of FTO thickness. The analysis of short-circuit current density, open-circuit voltage, fill factor, and solar cell efficiency, as a variable of FTO thickness from 100 nm to 600 nm are indicated in Fig. 3,while other layer values were kept constant. Fig. 3(a) indicates less change in open-circuit voltage and a slight decrease in short-circuit current density from 28.28 to 28.19 mA/cm^2^ with an increase in FTO thickness.

Fig. 3(b) indicates an increase of *FF* from 60.70% to 60.85% with an increment in FTO thickness from 100 nm to 600 nm and a slight reduction in efficiency from 16.17% to 16.10%. From the above, the decrease in efficiency by a small margin (0.070) has no much significance on the solar cell. An increase in FTO thickness from 100 nm to 600 nm does not have a significant effect, the thicker the layer, the more the absorption of photons. Therefore, 100 nm thick FTO was chosen to obtain the maximum *Voc* of the photovoltaic cell [22].

### 3.2 Impact of CdS thickness

**Fig. 4:**
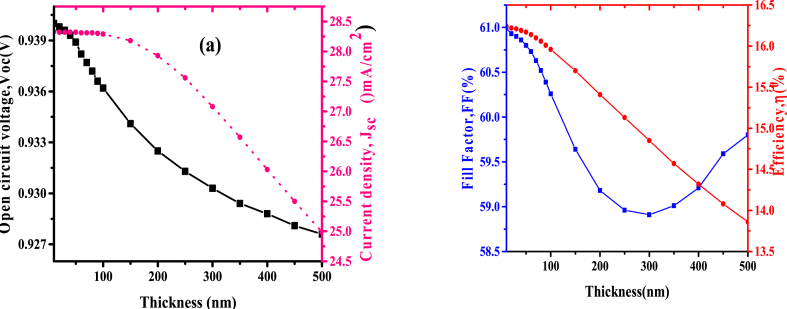
(a)Variation of *Voc* and *Jsc* (b) *FF* and efficiency with CdS thickness.

Cadmium sulphide has a major impact in operations of photovoltaic cell; transporting the generated electron from a photoactive layer to the front contact and hindering the hole from arriving at fluorine doped tin oxide electrode. The variation of Cadmium sulphide thicknesses were done from 10 nm to 500 nm while the thickness of Carbon were maintained at 50 nm, FTO at 100 nm, and CuSbS_2_ at 300 nm. From the simulation, both *Voc* and *Jsc* decrease with an increase in CdS thickness as elaborated in Fig. 4(a), also the fill factors and power conversion efficiencies reduced with the rise of the electron transport thickness layer as indicated in Fig. 4(b). Result implies that thicknesses increase causes charge recombination on ETL due to charges staying for a long time at the layer hence recombining with oppositely charged particles through the interface with the photoactive layer. Also, the increase of CdS in the path of incident solar radiation may have decreased transmittance therefore the delivered photons to CuSbS_2_ would have been less leading to low *Voc*. Because of the reduction in *Voc*, it also reduces the efficiency and the FF of the photovoltaic cell, this might have been a result of the rising of series resistance with increment in electron transport layer thicknesses. Therefore, optimum ETL thickness is 50 nm which gave the solar cell efficiency of 16.17%.

### 3.3 Effect of CuSbS_2_ thickness

**Fig. 5:**
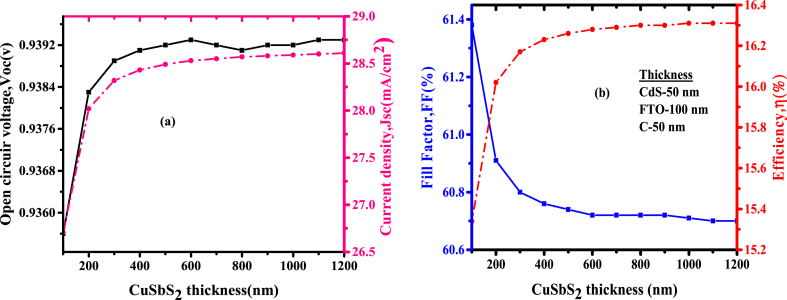
(a)Variation of *Voc* and *Jsc* (b) *FF* and efficiency with CuSbS_2_ thickness.

Thickness of photoactive layer determines the power conversion efficiency of the device, simulation of the photovoltaic cell was done to investigate the influence of CuSbS_2_ thickness on cell variables i.e. *Voc*, *Jsc*, *FF*, and efficiency. By maintaining other variable thicknesses constant, Variation of photoactive layer thickness was done from 200 nm to 1200 nm and considering that the absorber thickness must be greater than any other layer [18]. Fig. 5(a) depicts *Voc* and *Jsc* as dependent on CuSbS_2_ thickness and Fig. 5(b) illustrates *FF* and efficiency of the photovoltaic cell as a variable of CuSbS_2_ thickness. From Fig. 5(a) there is an increase of *Jsc* with the increment of FTO thicknesses, this is because of an increase of the absorbed radiations and carrier concentrations leading to a rise in *Jsc* as the thicknesses of the photoactive layer are increased. The increment in *Jsc* is related to a higher absorption coefficient of 10^5^ cm^−1^ of CuSbS_2_ layer [23]. It is also noted that there is a reduction in *Voc* when the absorber layer is above 700 nm, this results from recombination of charge carriers caused by the increment of absorber thickness resulting from near the center of the CuSbS_2_ layer which recombines when diffusion length exceeds the absorber thickness. In addition, the effective bandgap of copper antimony sulphide is decreased by the increase of its thickness leading to a reduction in *Voc* [17,18]. The fill factor slightly decreased from 61.38 to 60.70% as the thickness of CuSbS_2_ was increased. This is because the resistance increases with the increase in photoactive thickness resulting into reduced fill factor [24]. Also, due to an increment in recombination and a decline in extraction rate of charge carriers, the efficiency reduces slightly at a higher thickness. Therefore, an increment in photoactive thickness leads to the rate of absorption of long-wavelength solar irradiance to increase exciton generation and dissociation hence leading to more pairs of electron-hole thus the improvement of efficiency [9]. Therefore, the optimum thickness for the photoactive layer was 300 nm with a photovoltaic cell efficiency of 16.17%

### 3.4 Effect of carbon thickness

**Fig. 6:**
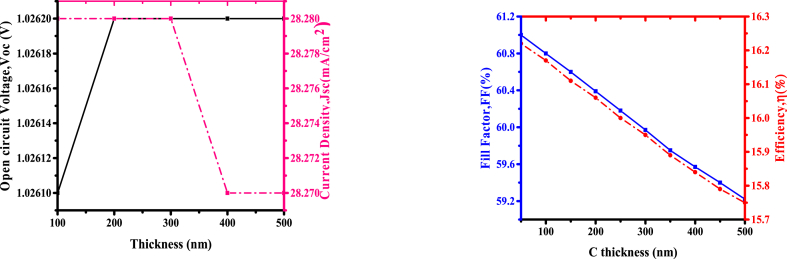
(a)Variation of *Voc* and *Jsc* (b) FF and efficiency with C thickness.

Carbon has a role in transporting the generated holes in the operations of the photovoltaic cells by blocking electrons [25]. While maintaining the thickness of CdS at 50 nm, CuSbS_2_ at 300 nm, and FTO at 100 nm variation of carbon thicknesses were done from 50 nm to 500 nm. From results obtained, Fig. 6(a) shows *Voc* and *Jsc* as a variable of C thickness while Fig. 6(b) indicates the *FF* and power conversion efficiency as variables of C thickness, an increment in HTL thickness caused a decline of *FF* and efficiency because of also a decline of irradiance and increase in recombination of charge carriers similar to work done by Hossain and his coworkers [26].

### 3.5 Effect of the doping concentration of CuSbS_2_ layer on photovoltaic cell parameters

**Fig. 7:**
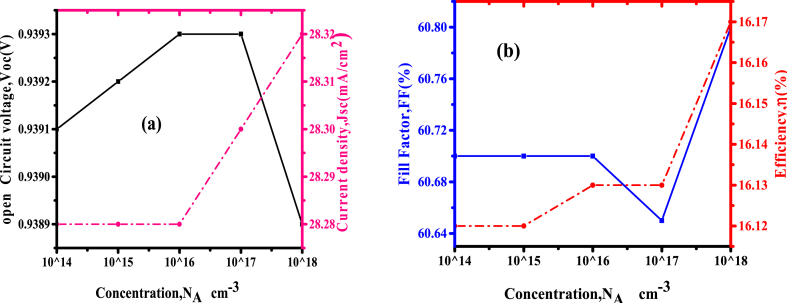
(a)Variation of *Voc* and *Jsc* (b) *FF* and efficiency with dopant concentration.

The electrical characteristics of the CuSbS_2_ materials are affected by its doping concentration which also influences the working of the photovoltaic cell. The effect of changing the doping concentration of the photoactive layer was done from 1 × 10^14^cm^−3^ to 1 × 10^18^ cm^−3^ to investigate the effect on solar cell parameters while absorber thickness was maintained at 300 nm. Results of the varied doping concentrations are indicated in Fig. 7.

The solar cell efficiency is more than 16% and the current density is highest at the doping concentration of 10^18^cm^−3^as shown above in Fig. 7(b). The *Voc* increases steadily as the concentration increases as shown in Fig. 7(a), because of the effective transport of charge carriers, although, at a doping concentration of 1 × 10^17^cm^−3^ the fill factor reduced slightly because of the reduction in ratio amongst the rates of recombination and generations of charge carrier in the major part of absorber layer as reported by Nalianya et al. [20]; the reduction of recombination rate is caused by an increment generation rate of free charges and increase in doping concentration, hence the *FF* starts to rise again, this is due to in p-type section there is an increase of majority carriers with the raising of acceptor dopant of copper antimony sulphide materials. Because high concentration leads to more auger recombination resulting in to increase in forward biasing.

### 3.6 Effect of defect density of absorber layer on solar cell parameters

The effect of the increase in defects density of the photoactive layer ware analyzed from 1 × 10^8^ cm^−3^ to 1 × 10^10^ cm^−3^ while maintaining the cross-sectional area at 1 × 10^−19^ cm^−2^. An increment in defects density caused increment of charge carrier recombination that resulted in a reduction of carrier lifetime and a decrease in diffusion length as shown in Table 2. Carrier lifetime increased when defects density reduced, leading to diffusion lengths becoming longer because of few recombination.

**Table 2:** The relationship between defect density and diffusion length.

Parameter			
Defect density (cm^−3^)	10^10^	10^9^	10^8^
Diffusion Length (nm)	719.53	2275.0	7195.3

### 3.7 J-V characteristics of the optimized parameters

**Fig. 8:**
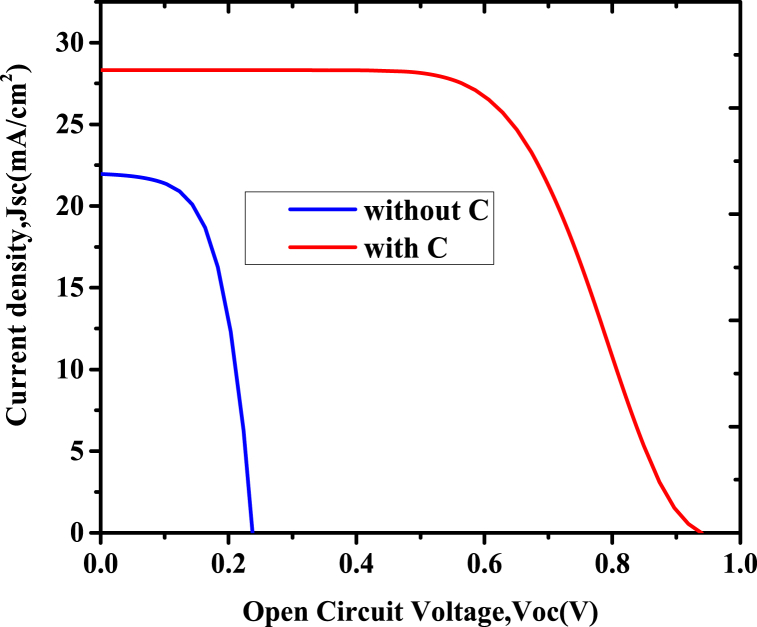
J–V curve of optimized solar cell with and without HTL.

The simulation result in Fig. 8 indicates that copper antimony sulphide has the ability to be an absorber material for application in photovoltaics because it shows a *Voc* of 0.9389 V, *Jsc* of 28.32 mA/cm^−2^, *FF* of 60.8%, and power conversion efficiency of 16.17% while using carbon as HTL. The J-V characteristic of optimized film without carbon depicted the open-circuit voltage of 0.2419 V, *Jsc* of 28.22 mA/cm^−2^, *FF* of 44.47 %, and efficiency of 3.4%.

## 4 Conclusion

CuSbS_2_ solar cell with design glass/SnO_2_: F/CdS/CuSbS_2_/C/Au was simulated vividly using SCAPS-1D software. The research on the impact of variation of layers thicknesses of FTO, CdS, CuSbS_2,_ and C was done on the device performances. From the simulations, CdS thickness variations have no much changes in the cell performance and the photoactive layer thickness of 300 nm–400 nm is appropriate for the workings of the device hence thicker photoactive layer may not have many benefits. Further, the thicknesses of 100 nm, 50 nm, 300 nm, and 100 nm for FTO, CdS, CuSbS_2_, and C layers respectively, revealed a better solar cell parameter. The variation of dopant concentration revealed that an increase in dopant concentration led to an increment in efficiency of the photovoltaic cell to 16.17%. Defect density increase led to a decrease in diffusion length indicating augmented recombination rate. Therefore, the optimized solar cell gave an efficiency of 16.17% suggesting good material for fabrication for photovoltaic applications.

## Data availability statement

Data is available in the supplementary file.

## CRediT authorship contribution statement

**Nancy Obare:** Writing – original draft. **Wycliffe Isoe:** Software. **Amos Nalianya:** Data curation. **Maxwell Mageto:** Supervision. **Victor Odari:** Supervision.

## Declaration of competing interest

I Nancy Obare.

Declare that we have no known competing financial interests or personal relationships that could have appeared to influence the work reported in this paper.
